# Femoral quadriceps neuromuscular electrical stimulation after total knee arthroplasty: a systematic review

**DOI:** 10.1590/S1679-45082015RW3140

**Published:** 2016

**Authors:** Helena Bruna Bettoni Volpato, Paulo Szego, Mario Lenza, Silvia Lefone Milan, Claudia Talerman, Mario Ferretti

**Affiliations:** 1 Hospital Israelita Albert Einstein, São Paulo, SP, Brazil.

**Keywords:** Arthroplasty, replacement, knee, Knee prosthesis, Knee joint, Osteoarthritis, Physical therapy modalities, Electric stimulation, Quadriceps muscle

## Abstract

The purpose of this study was to evaluate the effects of neuromuscular electrical stimulation in patients submitted to total knee arthroplasty. This was a systematic review with no language or publication status restriction. Our search was made in Cochrane Library, MEDLINE, Embase and LILACS. Randomized or quasi-randomized clinical trials evaluating neuromuscular electrical stimulation after total knee arthroplasty were included. Four studies with moderate risk of bias and low statistical power were included, totalizing 376 participants. There was no statistically significant difference in knee function, pain and range of motion during 12 month follow-up. This review concluded that neuromuscular electrical stimulation was less effective than traditional rehabilitation in function, muscular strength and range of motion. However, this technique was useful for quadriceps activation during the first days after surgery.

## INTRODUCTION

Osteoarthritis is a degenerative joint disease characterized by reduction of articular cartilage in some areas, and can be related to bone hypertrophy (osteophytes and subchondral bone sclerosis) resulting from biochemical alterations and biomechanical stresses. It is estimated that 75% of the population aged over 65 years is affected by this disease, with a higher prevalence in women, mostly at the knee joint.^1,2^

All the above-mentioned alterations result in quality of life reduction due to functional limitation. Knee arthroplasty is a common and effective intervention for knee osteoarthritis treatment when the clinical therapy is unsuccessful. Its applicability grew 73% at the last 10 years and it is expected that its indication rises more than 600% (3.48 million procedures) until 2030.^3^

Such percent growth in total knee arthroplasty (TKA), first introduced in the 1960s, is due to pain relief and range of motion (ROM) gain. TKA main goal is to reestablish the patient’s joint compromised function. This is why TKA is considered as one of the most important surgery developments of this century. On the United States alone, currently nearly 140 thousand TKAs are performed each year. However, surgery itself is not capable to restore the patient’s complete functionality. Physical therapy is an integral part of treatment, yielding the best results regarding postoperative pain, physical function and quality of life.^4^

As such, early rehabilitation start following TKA significantly benefits joint mobility and muscle stretch gain, favoring important quality of life gains and preventing postoperative complications. Apart from the primary benefits, the immediate rehabilitation could reduce hospital stay and improve the joint functional ability in the short and medium-run.^5^ Another study showed joint pain relief and gait velocity and cadence improvement.^6^

Labraca et al*.* ^2^ showed that, despite the few scientific evidences, isometric and isotonic exercises designed for quadriceps strengthening between zero and 30° of flexion, ROM gain and inferior limb muscle stretching are usually employed with good results.

Other studies showed that postoperative weakness, muscle atrophy and knee function alterations are common during the first 4 weeks after surgery, causing a quadriceps strength *déficit* compared to the contralateral limb that reaches a 18% reduction.^7,8^

Although the neurophysiologic mechanisms for quadriceps muscle voluntary activation *déficits* are not fully understood, spinal reflex activity from swelling or pain in the knee joint may change afferent input from the injured joint and result in diminished efferent motor drive to the quadriceps muscle (also referred to as “reflex inhibition”) that reduces muscle strength.^9,10^

One of the measures to reduce voluntary activation *deficits* and prevent muscle atrophy after TKA is the neuromuscular electrical stimulation (NMES) as an adjuvant restoring normal knee function.^9^

## OBJECTIVE

Based on the previously mentioned literature, the objective of this review was to systematically evaluate the effects (benefits and harms) of neuromuscular electrical stimulation in patients who underwent to total knee arthroplasty.

## METHODS

### Data sources and searches

Institutional review board approval (number 1,593-12) was obtained to perform this systematic review. The study was registered at - International Prospective Register of Systematic Reviews (Prospero), protocol CRD42013005491.

### Types of studies

We included randomized or quasi-randomized (in which participants therapy-allocation was not strictly random, *i.e.*, using hospital register number, alternation, medical file number etc.) clinical trials evaluating physical therapy interventions with NMES after TKA.

### Types of participants

We included studies that evaluated (skeletally mature) adults who underwent rehabilitation with NMES after TKA.

### Types of intervention

All physical therapy interventions used at post-TKA treatment associated with NMES were considered. We compared NMES with physical therapy rehabilitation.

Studies comparing non-physical therapy-related or specific formation rehabilitation techniques (including Mulligan, Maitland, Pilates etc.) were excluded.

### Types of outcome measures

Primary outcomes included function or disability evaluation. The inferior limb functional outcome was evaluated according validated questionnaires, such as the knee dysfunction-specific Lysholm Knee Scoring Scale,^11^ including joint symptoms evaluation.

Quality of life (evaluated by 36-Item Short Form Health Survey − SF-36),^12^ and treatment failure (prosthesis loosening) were also observed.

Secondary outcomes included pain, with pain reports evaluated according to validated scales, such as Visual Analogue Scale (VAS) or Numerical Rating Scale (NRS). Range of motion was checked by assessing the joint mobility degrees for both knee flexion and extension. Return to previous activities (work, sport, daily life activities etc.), as well as hospitalization time and costs were considered part of secondary outcomes.

Primary outcomes were evaluated at the following periods: short-term (up to 4 months of treatment) and long-term (over 4 months of treatment) follow-up.

### Research methods for studies identification

#### Electronic searches

Our search was performed at Cochrane Central Register of Controlled Trials - CENTRAL (The Cochrane Library, August 2013 edition), MEDLINE (from 1966 up to August 2013), Embase (from 1974 up to August 2013) and LILACS (from 1982 up to August 2013). We also searched Current Controlled Trials (at http://www.controlled-trials.com) and Clinical Trials (at www.clinicaltrials.gov) for ongoing and recently completed studies. There were no language or publication status-based restrictions.

At MEDLINE (PubMed), a specific filter (sensitivity and maximum precision version) for randomized clinical trials identification was combined to a specific subject strategy.^13^ Search strategies were also performed at The Cochrane Library (Wiley InterScience), Embase (Elsevier) and LILACS (Bireme) as described in appendix 1.

#### Searching other resources

We checked the reference lists of articles and reviews for possible relevant studies.

## Study selection

Two authors independently selected potential eligible titles and abstracts to be included on this review and extracted data using pre-piloted form. Any disagreements were resolved by discussion and, when necessary, with adjudication by a third author. Authors were not blinded to journal and/or authors.

## Data extraction and quality assessment

### Data extraction and management

Two authors collected the following data using a pre-piloted data extraction form: study methodology characteristics, including study design and duration and the protocol publication prior patient recruitment; financing sources and register details; study participants characteristics, with study site, number of enrolled participants, number of evaluated participants, inclusion criteria, exclusion criteria, participants’ age, prosthesis types and surgical techniques; study intervention characteristics, including intervention time, physical therapy intervention and other co-intervention types; study result characteristics, including follow-up time, loss at follow-up and outcome measures; and methodological domains, as described below at the risk of bias evaluation section. Any discrepancies were settled by a third reviewer. Two review authors inputted the data at Review Manager^TM^.

### Assessment of risk of bias in the included studies

The risk of bias of the included studies was independently evaluated by two authors. As recommended by The Cochrane Collaboration’s “Risk of bias” tool,^14^ the following domains were assessed: random sequence generation; allocation concealment; blinding of participants and personnel; blinding of outcome assessment; incomplete outcome data; selective reporting; other bias (*e.g.*, great imbalance between participants groups and risk of bias associated with testers and other caretakers’ inexperience).

Each individual criterion was deemed as presenting low risk of bias, high risk of bias and uncertain risk of bias (lack of information or uncertainties regarding potential bias). Discrepancies between authors were solved based on a consensus.

## Data synthesis and analysis

### Measures of treatment effect

The risk ratio with a 95% confidence interval (95%CI) was calculated for dichotomous variables. Continuous variables results were expressed as mean differences (MD) with 95%CI.

### Unit of analysis issues

At the studies included in this review, randomization was based on individual participants. Exceptionally, as in clinical trials including patients with bilateral knee prostheses, data may have been laterally evaluated rather than by individual patients. During the analysis of questions lacking proper corrections, the presentation of such clinical trials data was considered only when discrepancies between analysis units and randomization were small. After data compilation, a sensitivity analysis was performed to examine the effects of the incorrectly evaluated clinical trials in the studies correctly addressed.

### Dealing with missing data

The data on outcome were extracted for all randomized patients. When required, the primary study authors were contacted to request missing data, with participant number, sampling loss details, uncertainty measures (standard deviation or error) or number of events.

The standard deviation of continuous variables, with no report of such figure, was calculated using p value and values (95CI%).^14^ The impossibility of sampling loss data obtainment was described at the risk of bias table, including a discussion regarding the potential influence of such data at the results and conclusions of the present review. Sensitivity analysis was applied in order to explore these missing data effects.^15)^

## Assessment of heterogeneity

We assessed the heterogeneity of estimate effects between the included studies by visual inspection of the forest plot and using the I^2^ statistic.

We quantified the possible magnitude of inconsistency (*i.e.* heterogeneity) across studies, using the I^2^ statistic with a rough guide for interpretation as follows: zero to 40% might not be important; 30% to 60% may represent moderate heterogeneity; 50% to 90% may represent substantial heterogeneity; and 75% to 100% considerable heterogeneity.^14^ In cases of considerable heterogeneity (defined as I^2^ 75%), we explored the data further by comparing the characteristics of individual studies and conducting subgroup analyses.

### Assessment of reporting biases

For meta-analysis with more than ten studies, primary outcomes graphs were draw in order to evaluate the potential publication bias (small studies effects). The presence of bias was also evaluated in small studies, to verify if random intervention events were more beneficial compared to fixed events estimative.^16^

### Data synthesis

When appropriate, results of comparable groups of studies were pooled in meta-analysis using the random-effects model as a default. For dichotomous outcomes, RR and 95%CI were calculated. When two or more studies presented continuous data from the same validated instrument of evaluation using the same units of measure, data were pooled as a MD with 95%CI. When primary studies state the same variable using different instruments and different units of measure, we used the standardized mean difference with 95%CI.

### Subgroup analysis and investigation of heterogeneity

Subgroup analysis for following demographics was planned: age (adolescents, adults and people older than 65); type of surgical intervention TKA; and rehabilitation start (outpatient and inpatient).

### Sensitivity analysis

We planned sensitivity analyses to measure the effects of including trials at risk of selection bias (inadequate or unclear allocation concealment) or detection bias (inadequate or unclear blinding of outcome assessor). We also planned to assess the presence of small study bias (*i.e.* intervention effect is more beneficial in smaller studies) in the meta-analysis by comparing the fixed-effect estimate with the random-effects estimate for primary outcomes.

### ‘Summary of findings’ tables and assessment of the quality of the evidence

When there is sufficient evidence in future to merit the preparation of summary of findings tables, we will develop these for the main comparisons. We used the GRADE approach to assess the quality of evidence related to each of the key outcomes listed in the types of outcome measures.^17^

## RESULTS

### Search results

The search strategy (concluded in August 2013) identified a total of 584 registers at the following current databases: Cochrane Library (129), PubMed (166), Embase (143), LILACS (130), Clinical Trials (11) and Controlled Trials (5).

The search led to the identification of 18 potentially eligible studies, from which the complete papers were retrieved. A total of four studies, published between 1994 and 2013, were included on the review.^18-21^

**Figure 1:**
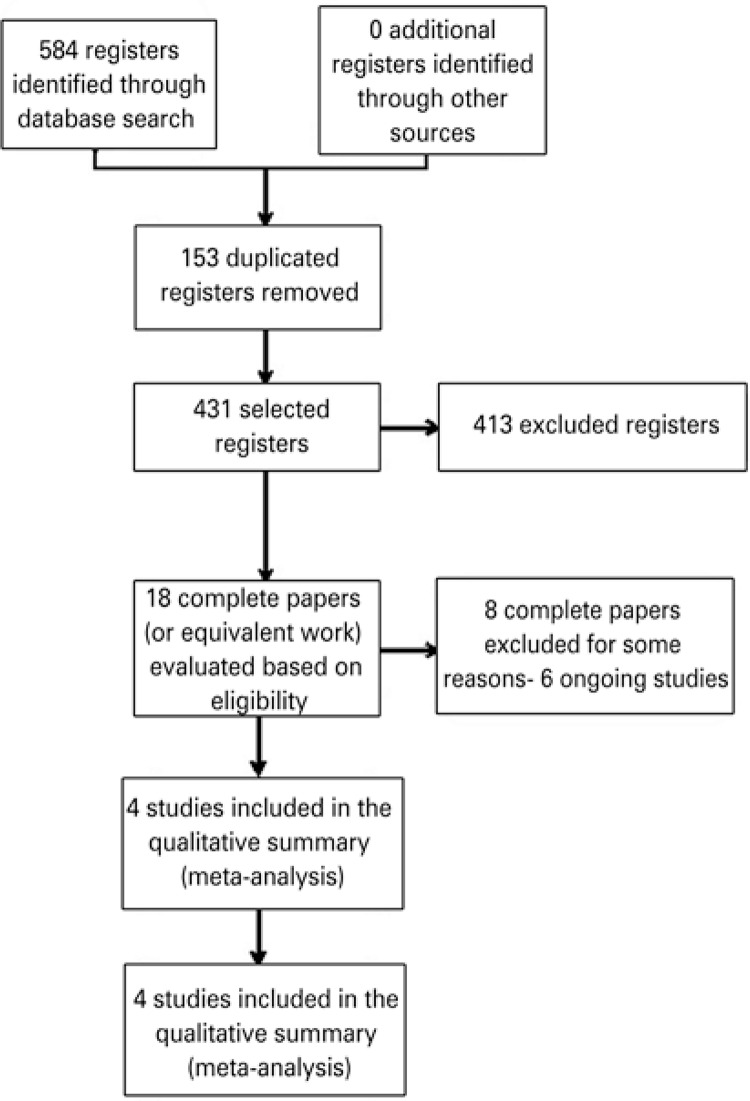
Study flowchart

Overall, there were four included studies, eight excluded studies and six ongoing studies (Figure 1).

### Included studies

This review was based on four randomized clinical trials: Gotlin et al.,^18^ Levine et al.,^19^ Petterson et al.^20^ and Stevens-Lapsley et al.^21^ (all studies presented in English). These trials were found at PubMed, Cochrane Library and Embase. Included studies details are specified at appendix 2.

### Study design

Gotlin et al.,^18^ Levine et al.,^19^ Petterson et al.^20^ and Stevens-Lapsley et al.^21^ were single-center controlled randomized studies. All studies compared two groups undergoing the same interventions (exercises *versus* exercises and NMES). Gotlin et al*.* ^18^ evaluated exercises and continuous passive motion (Control Group) *versus* exercises, continuous passive motion and NMES (experimental group).

### Participants

The four studies included trials totalized 376 participants.

### Age and sex

Gotlin et al.^18^ described that mean age was 64.8 years in the exercise group (control) and 66.2 years in the NMES plus exercise group (intervention). The Control Group comprised 16 women and 15 men, and the intervention group comprised 20 women and 15 men. In Levine et al*.*,^19^ the mean age was 65.1 years in the Control Group, and 68.1 years in the intervention group. The Control Group comprised 21 women and 13 men, and the intervention group was composed of 25 women and 7 men. Petterson et al*.* ^20^ reported that the Control Group were composed of 45 woman and 55 were men with mean age of 65.2 years-old. In the intervention group, 47 participants were women and 53 were men, presenting mean age between sexs of 65.3 years-old. In Stevens-Lapsley et al.,^21^ the sample consisted of 16 women and 15 men in the Control Group and 20 women and 15 men in the intervention group, presenting mean age between sexs of 64.8 years-old for the Control Group and 66.2 years-old for the intervention group.

All participants, in all trials, held a unilateral or bilateral knee osteoarthritis diagnosis. None of the studies described the classification or TKA previous treatment.

### Interventions

The included studies were grouped according to the interventions analyzed.

Gotlin et al*.* ^18^ analyzed the effects of exercises and continuous passive motion (Control Group) *versus* exercises, continuous passive motion and NMES (experimental group) in 40 patients. Levine et al*.* ^19^ compared a muscle strengthening program and knee ROM gain *versus* NMES associated only to knee ROM exercises in 70 participants. Petterson et al*.* ^20^ and Stevens-Lapsley et al*.* ^21^ compared a progressive muscle (quadriceps) strengthening program *versus* NMES associated with progressive muscle strengthening, both of early start (immediate postoperative), in 266 participants.

### Primary outcomes

#### Function or deficiency

Knee function was evaluated in three studies.^19-21^ Levine et al*.* ^19^ used Timed Up and Go (TUG), Western Ontario and McMaster Universities Osteoarthritis Index (WOMAC) questionnaire and Knee Society Score (KSS) to measure the joint function. Petterson et al.^20^ and Stevens-Lapsley et al.^21^ used validated instruments − Timed Up and Go (TUG) Stair Climbing Test (SCT), 6-Minute Walk (6MW) − to evaluate knee functional compromise. Stevens-Lapsley et al.^21^ also used the WOMAC questionnaire. In addition to these instruments, Petterson et al.^20^ and Stevens-Lapsley et al.^21^ evaluated quadriceps muscle strength using dynamometry.

#### Quality of life

Gotlin et al*.* ^18^ and Levine et al.^19^ did not measure this endpoint. Petterson et al.^20^ and Stevens-Lapsley et al.^21^ evaluated the quality of life using SF-36.

#### Treatment failure

All included trials reported no treatment failures.

## Secondary outcome

### Pain

Pain was evaluated in three studies.^19-21^ Levine et al.^19^ used the validated score from KSS to measure pain, and Petterson et al.^20^ used a validated score *(*Knee Outcome Survey - KOS and Activities of Daily Living Scale *-* ADLS); Stevens-Lapsley et al.^21^ measured individuals pain complaint applying a numeric visual scale.

### Range of motion

In Gotlin et al.,^18^ Petterson et al*.* ^20^ and Stevens-Lapsley et al.^21^ studies were evaluated through goniometry. Levine et al.^19^ also evaluated their participants’ knee ROM, but did not report which instrument was used to measure this outcome.

## Excluded studies

Eight papers were excluded for not meeting the inclusion criteria. The reasons for exclusion are presented in chart 1.

**Chart 1:** Characteristics of the excluded studies

Study	Reason for exclusion
Petterson et al.^(22)^	Case report
Mintken et al.^(23)^	Case report
Stevens et al.^(9)^	Case series
Stevens-Lapsley et al.^(24)^	Observational study
Bade et al.^(25)^	Literature review
Saleh et al.^(26)^	Literature review
Walls et al.^(27)^	NMES use only in the pre-operative period
Stevens et al.^(28)^	Incomplete unpublished data

NMES: neuromuscular electrical stimulation.

## Ongoing studies

Our search for ongoing studies resulted in 16 papers on Clinical Trials and Current Controlled Trials. Ten studies were excluded for not meeting our inclusion criteria or being irrelevant. We included: ISRCTN89785408, ISRCTN50117467, NCT01096524, NCT01548040, NCT00224913 and NCT01844193 (Appendix 3).

## Risk of bias in the included studies

**Figure 2:**
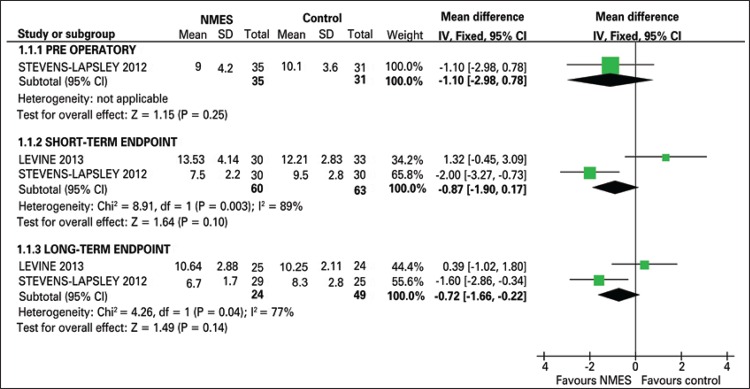
Risk of bias chart

All included trials had methodological flaws, rendering them at moderate risk of bias (Figure 2).

NMES:neuromuscular electrical stimulation. SD: standard deviation; IV:inverse variance; 95%CI: 95% confidence interval.

## Allocation (selection bias)

Gotlin et al.,^18^ Levine et al*.*,^19^ Petterson et al*.* ^20^ and Stevens-Lapsley et al*.* ^21^ did not report how the random sequence generation was performed.

Concealment of allocation before assignment was not described in any study, preventing its appreciation (unclear).

## Blinding (performance bias and detection bias)

All trials were judged to be at high risk of performance and detection bias. As they all compared physical therapy interventions, it was not possible to blind treatment providers. No trials included sham intervention; therefore participants were not blinded. It may have been possible to blind outcome assessors; however, only two trials mentioned assessor blinding.^18-20^

## Incomplete outcome data (attrition bias)

Trials with 80% or more participants completing follow-up and those whose losses were balanced between intervention groups were deemed as presenting low risk of bias. All included trials^18-21^ were considered as presenting low risk of bias.

## Selective report (reporting bias)

Three studies^19-21^ were considered as presenting low risk of bias, since their protocols and pre-specified outcomes were available. On the other hand, Gotlin et al*.* ^18^ was considered as high risk of bias, since it did not present a specified protocol.

## Additional potential bias sources

Gotlin et al*.*,^18^ Petterson et al*.* ^20^ and Stevens-Lapsley et al*.* ^21^ trials seem to be free from additional biases. Only Levine et al*.* ^19^was considered as high risk of bias, since it not presented a NMES protocol.

## Effects of interventions

The included studies evaluated the following outcomes: function or disability, ROM, quality of life and treatment failure. Additional outcomes planned in our protocol were not evaluated due to insufficient data.

### Comparison

Neuromuscular electrical stimulation *versus* exercises (with or without continuous passive motion) starting up to the first postoperative week.

## Function or disability

Function measures were analyzed and made available in the following sequence: TUG, 6MW, SCT, quadriceps activation, femoral quadriceps strength, and WOMAC.

### Timed Up and Go

**Figure 3:**
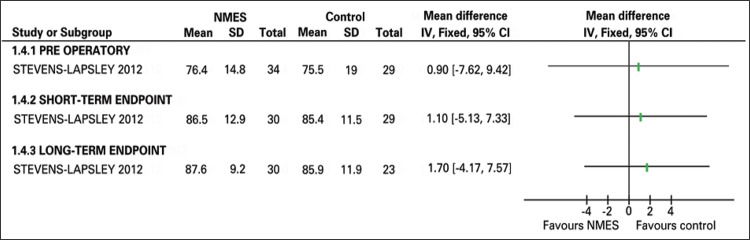
Timed Up and Go graph NMES: neuromuscular electrical stimulation. SD: standard deviation; IV: inverse variance; 95%CI: 95% confidence interval.

In Levine et al*.*,^19^ there were no significant differences in both the short-term (MD: 1.32; 95%CI: -0.45-3.09) and long-term endpoint (MD: 0.39; 95%CI: -1.02-1.80). In the Stevens-Lapsley et al*.* ^21^ study, we noted a statistically significant difference favoring NMES in both the short-term (MD: -2.00; 95%CI: -3.27- -0.73) and long-term endpoint (MD: -1.60; 95%CI: -2.86- -0.34). Studies data grouping did not show any statistically significant difference between the two intervention groups (MD: -0.87; 95%CI: -1.90-0.17), (MD: -0.72; 95%CI: -1.66-0.22) at short-term and long-term endpoint, respectively (Figure 3).

Petterson et al*.* ^20^ reported no statistically significant difference (p>0.08).

## 6-minute walk test

Difference between groups in Stevens-Lapsley et al*.* ^21^ study marginally favored control at the short-term endpoint (MD: 62.60; 95%CI: 15.51-109.69). At long-term endpoint, no difference between groups was noted (MD: 46.80; 95%CI: -0.53-94.13). No statistically significant difference was reported by Petterson et al.^20^ (p>0.08).

### Stair Climbing Test

Petterson et al*.* ^20^ report no significant difference for this outcome at the short-term or long-term endpoint (p>0.08). At Stevens-Lapsley et al*.* ^21^, no statistically significant difference between the two groups - NMES and exercises (MD: -2.70; 95%CI: -6.40-1.00), (MD: -3.30; 95%CI: -7.27-0.67) was noted in both short-term and long-term endpoint, respectively.

### Quadriceps activation (%)

**Figure 4:**
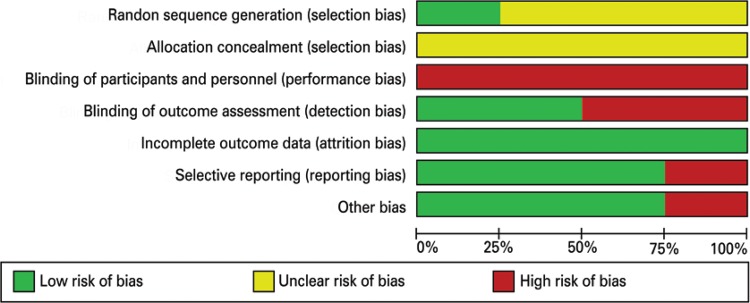
Quadriceps activation chart

Petterson et al*.* ^20^ reported the same, with p>0.08. Stevens-Lapsley et al.^21^ reported no statistically significant difference at the short-term endpoint (MD: 1.10; 95%CI: -5.13-7.33) between both groups and at the post-TKA short-term endpoint (MD: 1.70; 95%CI: -4.17-7.57) (Figure 4).

### Femoral quadriceps strength (N-m/kg)

Petterson et al*.* ^20^ also reported the lack of significant difference between groups (p>0.08). No statistically significant differences between the two groups at the short-term (MD: 0.22; 95%CI: -0.02-0.46) and the long-term endpoint (MD: 0.16; 95%CI: -0.09-0.41) were noted at Stevens-Lapsley et al*.* ^21^

## Quality of life

The SF-36 questionnaire was used to measure the quality of life during and after the physical therapy intervention in both Petterson et al*.* ^20^and Stevens-Lapsley et al*.* ^21^studies and data were analyzed according to its components.

### SF-36 physical component summary

Petterson et al*.* ^20^ reported no significant difference between groups (p>0.08). Stevens-Lapsley et al.^21^ also reported no statistically significant differences at the short-term (MD: 3.20; 95%CI: -1.50-7.90) and the long-term endpoint (MD: 1.90; 95%CI: -1.97-5.77).

### SF-36 mental component summary

Petterson et al*.* ^20^ reported no significant difference, with p>0.08. At the mental component summary, Stevens-Lapsley et al*.* ^21^ also reported no statistically significant difference in both the short-term (MD: 3.70; 95%CI: -1.55 -8.95) and long-term endpoint (MD: 3.00; 95%CI: -0.39 -6.39).

### Quality of life and function

WOMAC questionnaire was used by Levine et al*.* ^19^ to measure knee function, with no statistically significant difference between groups in both the short-term (MD: 5.67; 95%CI: -1.88-13.22) and long-term endpoint (MD: 5.81; 95%CI: -3.01-14.63). Stevens-Lapsley et al.^21^also reported no statistically significant difference in both the short-term (MD: -6.80; 95%CI: -15.04-1.44) and at the long-term endpoint (MD: -4.30; 95%CI: -9.99-1.39). Data pooling showed no statistically significant difference in both the short-term (MD: -0.02; 95%CI: -5.59-5.54) and at the long-term endpoint (MD: -1.33; 95%CI: -6.11 -3.45) between the two intervention groups.

Levine et al*.* ^19^ used the KSS questionnaire to check knee function and reported no statistically significant difference in both the short-term (MD: 4.97; 95%CI: -4.65-14.59) and the long-term endpoint (MD: 7.92; 95%CI: -2.29-18.13).

Petterson et al*.* ^20^ used the KOS questionnaire to measure knee function and also reported no statistically significant difference in both short-term and long-term endpoint (p>0.01).

## Treatment failure

All included trials reported no treatment failures.

## Pain

None of the included studies individually evaluated this outcome.

## Range of motion

### Knee flexion

Levine et al*.* ^19^ reported no statistically significant difference at short-term (MD: -3.20; 95%CI: -8.52 -2.12) and long-term endpoint (MD: 2.30; 95%CI: -4.25 -8.85). Stevens-Lapsley et al*.* ^21^ reported no statistically significant difference between groups - NMES and exercises (MD: 2.90; 95%CI: -1.89-7.69 and MD: 2.40; 95%CI: -1.82-6.62) at the short-term and long-term endpoint, respectively. Petterson et al.^20^ also reported no statistically significant difference (p>0.01). Data pooling showed no statistically significant difference between the two intervention groups (MD: 0.17; 95%CI: -3.39-3.73; and MD: 2.37; 95%CI: -1.18-5.92) in both the short-term and long-term endpoint, respectively.

### Knee extension

Gotlin et al*.* ^18^ and Stevens-Lapsley et al*.* ^21^ reported a statistically significant difference favoring the intervention group compared with the group without intervention in the short-term endpoint (MD: -2.65; 95%CI: -4.05- -1.25 *versus* MD: -2.40; 95%CI -4.09- -0.71) respectively, with no statistically significant difference in the long-term endpoint, that was reported only in the study by Stevens-Lapsley et al*.* ^21^(MD: -0.60; 95%CI: -2.43-1.23). Levine et al.^19^ reported no significant difference in both the short-term endpoint (MD: 0.65; 95%CI -2.00 -3.30) and the long-term endpoint (MD: -0.96; 95%CI: -3.62-1.70). When data were pooled, a statistically significant difference in the short-term endpoint was noted (MD: -2.09; 95%CI: -3.09- -1.09); however, there was no statistically significant difference in the long-term endpoint (MD: -0.72; 95%CI: -2.22-0.79) in both intervention groups.

Petterson et al.^20^ report no significant difference between both groups during all months of intervention (p>0.01).

## DISCUSSION

At this review, only randomized clinical trials were included, leading to the analysis of four trials deemed of moderate risk of bias and evaluating kinesiotherapic physical therapy interventions compared to NMES with physiotherapy use in 376 participants undergoing TKA.

No evidence indicated if NMES with physiotherapy provided benefits regarding the quality of life. The postoperative treatment with NMES can improve the femoral quadriceps function, but we are not sure about the effectiveness of this intervention, due to the low quality evidence.

Very low evidence from the included trials presented a low general quality resulting from methodological failures, including the lack of allocation concealment and participants and personnel blinding in all trials. However, the quantitative results of this review must be carefully interpreted, requiring confirmation of such data by evidence derived from high methodological quality trials.

We believe that our search strategy was complete, with no language restriction. However, it is possible that we missed some potentially eligible studies. We tried to contact the included studies’ authors in order to obtain some data, but with no success.

We found a systematic review comparing NMES *versus* exercise therapy to treat the quadriceps inhibition after TKA (Monaghan et al).^29^ including two randomized and non-randomized clinical trials, and a total of 69 subjects. Our study results are consistent with the Cochrane systematic review results and our conclusion is similar to this publication.

Monaghan et al.^29^ evaluated NMES use for quadriceps strengthening pre-and post-TKA and reported no significant differences for these outcomes; however, they considered included studies with a high risk of bias due to study design limitations and presented results imprecision, preventing a meta-analysis performance. The authors concluded that the identified studies do not allow any definition regarding NMES pre- or post-TKA.

Monaghan et al.^29^ also interpreted that participants undergoing quadriceps NMES presented a slight advantage in function improvement and less deficiency than those conservatively treated in the short follow-up, just as we see at the clinical practice and consistent with the findings in Gotlin et al.^18^ However, efficacy was reduced at the long-term follow-up.

Therefore, this review is inconclusive about NMES efficacy, and further evidence is required to support or deny its use at quadriceps activation after TKA. The authors are aware that this review subject is the object of an ongoing investigation and could be updated in order to incorporate new evidences.

## CONCLUSION

The very low evidences from included studies found on this review do not allow any conclusions regarding neuromuscular stimulation application for quadriceps strengthening with physical therapy before or after total knee replacement. Until now, evidence for neuromuscular stimulation use to quadriceps strengthening in this patient group is unclear. However, it is critical that future studies verify the quadriceps strength pre- and post-neuromuscular stimulation using reliable evaluations and validated tools, as well as a clear description of the applied dose in the study design. It is also of uttermost importance that results be presented appropriately for meta-analysis performance.
