# Supplementary material for: Femoral quadriceps neuromuscular electrical stimulation after total knee arthroplasty: a systematic review
Source: Einstein (Sao Paulo). 2016 Jan-Mar;14(1):77–98. doi: 10.1590/S1679-45082015RW3140 (PMC4872923; doi:10.1590/S1679-45082015RW3140)
Supplement: Translation

# Translation: Estimulação elétrica neuromuscular do quadríceps após artroplastia total de joelho: uma revisão sistemática

**Authors:** Helena Bruna Bettoni Volpato, Paulo Szego, Mario Lenza, Silvia Lefone Milan, Claudia Talerman, Mario Ferretti

**Affiliations:** 1 Hospital Israelita Albert Einstein, São Paulo, SP, Brasil.

**Keywords:** Artroplastia de joelho, Prótese do joelho, Articulação do joelho, Osteoartrite, Modalidades de fisioterapia, Estimulação elétrica, Músculo quadríceps

## Abstract

O objetivo deste estudo foi avaliar os efeitos da estimulação elétrica neuromuscular em pacientes submetidos à artroplastia total de joelho. Esta foi uma revisão sistemática sem restrição de idioma ou *status* de publicação. Nossa pesquisa foi realizada na biblioteca Cochrane, MEDLINE, Embase e LILACS. Foram incluídos ensaios clínicos randomizados ou quase-randomizados, avaliando a estimulação elétrica neuromuscular após a artroplastia total de joelho. Quatro estudos com risco de viés moderado e de baixo poder estatístico foram incluídos, totalizando 376 participantes. Não houve diferença estatisticamente significativa na função do joelho, dor e amplitude de movimento durante 12 meses de seguimento. Esta avaliação concluiu que a estimulação elétrica neuromuscular foi menos eficaz do que a reabilitação tradicional na função, força muscular e amplitude de movimento. No entanto, essa técnica é útil para a ativação do quadríceps durante os primeiros dias após a cirurgia.

## INTRODUÇÃO

A osteoartrose é uma doença articular degenerativa caracterizada pela redução da cartilagem articular em algumas áreas, podendo estar relacionada a hipertrofia óssea (osteófitos e esclerose óssea subcondral) resultante de alterações bioquímicas e de estresses biomecânicos. Estima-se que 75% da população com mais de 65 anos seja afetada por essa doença, com maior prevalência nas mulheres, na maioria dos casos na articulação do joelho.^(1,2)^

Todas as alterações acima mencionadas resultam em diminuição da qualidade de vida, devido à limitação funcional. A artoplastia do joelho é uma intervenção comum e eficaz para o tratamento da osteoartrose do joelho, quando o tratamento clínico falha. Sua aplicabilidade aumentou em 73% nos últimos 10 anos e espera-se que sua indicação aumente em mais de 600% (3,48 milhões de procedimentos) até 2030.^(3)^

Esse crescimento percentual da artroplastia total de joelho (ATJ), realizada pela primeira vez na década de 1960, deve-se ao alívio da dor e ao ganho de amplitude de movimento (ADM). O principal objetivo da ATJ é restabelecer a função comprometida do joelho do paciente. É por isso que a ATJ é considerada um dos avanços cirúrgicos mais importantes deste século. Somente nos Estados Unidos, são realizadas atualmente quase 140 mil ATJ a cada ano. No entanto, a cirurgia por si só não é capaz de restaurar a completa funcionalidade do paciente. A fisioterapia é uma parte integral do tratamento, apresentando os melhores resultados no tocante a dor, função física e qualidade de vida após a cirurgia.^(4)^

Como tal, o início precoce da reabilitação após a ATJ beneficia significativamente o ganho de mobilidade articular e extensibilidade muscular, favorecendo importantes ganhos de qualidade de vida e prevenindo complicações pós-operatórias. Além dos benefícios primários, a reabilitação imediata pode reduzir o tempo de internação e melhorar a capacidade funcional da articulação a curto e médio prazo.^(5)^ Outro estudo mostrou alívio da dor articular e melhoria na velocidade e na cadência da marcha.^(6)^

Labraca et al.^(2)^ mostraram que, apesar de haver poucas evidências científicas, os exercícios isométricos e isotônicos designados para fortalecimento do quadríceps entre zero e 30° de flexão, ganho de ADM e extensibilidade muscular do membro inferior são geralmente empregados com bons resultados.

Outros estudos mostraram que fraqueza, atrofia muscular e alterações funcionais do joelho são comuns nas 4 primeiras semanas após a cirurgia, causando um *déficit* de força do quadríceps em comparação ao membro contralateral, que chega a uma redução de até 18%.^(7,8)^

Embora os mecanismos neurofisiológicos das alterações na ativação muscular voluntária do quadríceps não sejam totalmente compreendidos, a atividade reflexa espinal decorrente do edema ou da dor na articulação do joelho pode mudar o processo de entrada aferente da articulação lesada e resultar na diminuição do impulso motor eferente para o músculo quadríceps (também chamado de “inibição reflexa”), que reduz a força muscular.^(9,10)^

Uma das medidas para reduzir as alterações na ativação voluntária e prevenir a atrofia muscular após a ATJ é a estimulação elétrica neuromuscular (EENM) como recurso adjuvante para restaurar a função articular normal do joelho.^(9)^

## OBJETIVO

Com base na literatura mencionada, o objetivo desta análise foi avaliar sistematicamente os efeitos (benefícios ou prejuízos) da estimulação elétrica neuromuscular nos pacientes submetidos à artroplastia total de joelho.

## MÉTODOS

### Fontes de dados e pesquisas

Obteve-se aprovação da junta de revisão institucional (número 1.593-12) para a realização desta revisão sistemática. O estudo foi registrado no *International prospectiveregister of systematic reviews* (Prospero), sob protocolo CRD42013005491.

### Tipos de estudo

Incluímos estudos clínicos randomizados ou quase-randomizados (nos quais a alocação da terapia dos participantes não foi estritamente randomizada, ou seja, usando-se número de registro hospitalar, alternância, número do prontuário etc.), ensaios clínicos que avaliavam as intervenções de fisioterapia com EENM após ATJ.

### Tipos de participante

Incluímos estudos que avaliavam adultos (esqueleticamente maduros) que se submeteram à reabilitação com EENM após ATJ.

### Tipos de intervenção

Todas as intervenções de fisioterapia usadas no tratamento pós-ATJ associadas com EENM foram levadas em consideração. Comparamos a EENM com a reabilitação fisioterapêutica.

Foram excluídos os estudos que comparavam técnicas não relacionadas a fisioterapia ou técnicas de reabilitação de formação específica (incluindo Mulligan, Maitland, Pilates etc.).

### Tipos de medidas de resultados

Os resultados primários incluíram a avaliação da função ou incapacidade. O resultado funcional do membro inferior foi avaliado de acordo com questionários validados, como a escala de avaliação funcional específica para joelho *Lysholm Knee Scoring Scale,* ^(11)^ incluindo a avaliação de sintomas articulares.

A qualidade de vida (avaliada pelo *36-Item Short Form Health Survey* − SF-36);^(12)^ e a falha do tratamento (soltura da prótese) também foram observados primariamente.

Os resultados secundários incluíram a dor, sendo os relatórios de dor avaliados de acordo com escalas validadas, como a *Visual Analogue Scale* (VAS) ou a *Numerical Rating Scale* (NRS). A ADM foi checada pelos níveis de mobilidade articular, tanto para a flexão quanto para a extensão do joelho. Retorno a atividades prévias (trabalho, esportes, atividades cotidianas etc.), e tempo e custo de hospitalização também fizeram parte dos resultados secundários.

Os resultados primários foram avaliados nos seguintes períodos: seguimento de curto prazo (até 4 meses de tratamento) e de longo prazo (acima de 4 meses de tratamento).

### Métodos de pesquisa para identificação de estudos

#### Pesquisas eletrônicas

Nossa pesquisa foi realizada nas bibliotecas *CochraneCentral Register of Controlled Trials -* CENTRAL *(TheCochrane Library*, edição de agosto de 2013), MEDLINE (de 1966 até agosto de 2013), Embase (de 1974 até agosto de 2013) e LILACS (de 1982 até 2013). Também pesquisamos a *Current Controlled Trials* (em http://www.controlled-trials.com) e o Ensaios Clínicos (em www.clinicaltrials.gov) para estudos em andamento e estudos concluídos recentemente. Não houve restrições de idioma e nem de *status* de publicação.

Na MEDLINE (PubMed), um filtro específico (versão de sensibilidade e precisão máxima) para a identificação de estudos clínicos randomizados foi combinado com uma estratégia de assunto específico.^(13)^ Também foram realizadas estratégias de pesquisa na *The Cochrane Library* (Wiley InterScience), na Embase (Elsevier) e na LILACS (Bireme), conforme descrito no anexo 1.

#### Pesquisa de outros recursos

Verificamos as listas de referências de artigos e revisões para localizar possíveis estudos relevantes.

#### Seleção de estudos

Dois autores selecionaram, de forma independente, os títulos e resumos potencialmente elegíveis para inclusão nesta revisão e extraíram os dados usando um formulário pré-testado. Todas as divergências foram resolvidas por consenso ou, quando necessário, por um terceiro autor. Não houve cegamento dos autores para as revistas e/ou autores.

## Extração de dados e avaliação de qualidade

### Extração e análise dos dados

Dois autores coletaram os seguintes dados usando um formulário de extração de dados pré-testado: características metodológicas do estudo, incluindo desenho e duração do estudo, e protocolo de publicação anterior ao recrutamento de pacientes; fontes de financiamento e detalhes de registro; características dos participantes do estudo, com local do estudo, número de participantes inscritos, número de participantes avaliados, critérios de inclusão, critérios de exclusão, idade dos participantes, tipos de prótese e técnicas cirúrgicas; características da intervenção do estudo, incluindo tempo de intervenção, intervenção de fisioterapia e outros tipos de cointervenções; características dos resultados do estudo, incluindo tempo de seguimento, perda de seguimento e medidas de desfecho; e domínios metodológicos, conforme descritos na seção de avaliação de riscos de viés. Todas as discrepâncias foram resolvidas por um terceiro revisor. Dois revisores inseriram os dados no *Review Manager* ^®^.

## Avaliação de risco de viés nos estudos incluídos

O risco de viés dos estudos incluídos foi avaliado independentemente pelos dois autores. Conforme recomendado pela ferramenta “Risco de viés”, da *The Cochrane Collaboration*,^(14)^ os seguintes domínios foram avaliados: geração de sequência aleatória; ocultação da alocação; cegamento dos participantes e da equipe; cegamento da avaliação de resultado; dados de resultado incompletos; relatório seletivo; (por exemplo, outros vieses, como grande desequilíbrio entre os grupos de participantes e risco de viés associado à inexperiência dos examinadores e de outros cuidadores).

Cada critério individual foi considerado como tendo baixo risco de viés, alto risco de viés ou risco de viés incerto (falta de informação ou incertezas referentes a vieses em potencial). As discrepâncias entre os autores foram resolvidas por consenso.

## Síntese e análise de dados

### Medidas do efeito do tratamento

A taxa de risco com intervalo de confiança de 95% (IC95%) foi calculada para as variáveis dicotômicas. Os resultados das variáveis contínuas foram expressos como diferença média (DM) com IC95%.

### Questões de unidade de análise

Nos estudos incluídos nesta revisão, a randomização baseou-se nos participantes individuais. Excepcionalmente, como nos estudos clínicos que incluíam pessoas com próteses de joelho bilaterais, os dados podem ter sido avaliados lateralmente em vez de por paciente individual. Durante a análise das questões que careciam das devidas correções, a apresentação dos dados desses estudos clínicos somente foi levada em consideração quando as discrepâncias entre as unidades de análise e a randomização foram pequenas. Depois da compilação dos dados, uma análise de sensibilidade foi realizada para avaliar os efeitos desses estudos clínicos analisados incorretamente nos estudos analisados corretamente.

### Manejo dos dados faltantes

Foram extraídos dados de resultados de todos os pacientes randomizados. Quando exigido, contatamos os autores do estudo primário para solicitar dados faltantes, como número de participantes, detalhes de perdas de amostra, medidas de incertezas (erro ou desvio padrão) ou número de eventos.

O desvio padrão das variáveis contínuas, para as quais esse valor não foi relatado, foi calculado usando os valores de p e do IC95%.^(14)^ A impossibilidade de obtenção dos dados de perdas de amostras foi descrita na tabela sobre risco de viés, incluindo um comentário sobre a influência em potencial desses dados nos resultados e conclusões desta revisão. A análise de sensibilidade foi aplicada para explorar o efeito desses dados faltantes.^(15)^

## Avaliação de heterogeneidade

Avaliamos a heterogeneidade dos efeitos estimados entre os estudos incluídos por meio de inspeção visual do gráfico de metanálise (*forest plot*) e pelo uso da estatística I^2^.

A magnitude possível de inconsistência (ou seja, heterogeneidade) ao longo dos estudos foi quantificada, usando a estatística I^2^ como guia aproximado para interpretação, como se segue: valores de zero a 40% talvez não sejam importante; de 30% a 60% podem representar heterogeneidade moderada; de 50% a 90% podem representar heterogeneidade substancial; de 75% a 100% indicam heterogeneidade considerável.^(14)^ Nos casos de heterogeneidade considerável (definida como I^2^ de 75%), exploramos mais os dados comparando as características dos estudos individuais e realizando análises de subgrupos.

### Avaliação dos relatórios de viés

Para metanálise com mais de dez estudos, foram feitos gráficos de resultados primários para avaliar o viés em potencial das publicações (efeito de estudos de pequeno porte). A presença de viés também foi avaliada nos estudos menores para verificar se os eventos de intervenção aleatórios eram mais benéficos quando comparados à estimativa de eventos fixos.^(16)^

### Síntese de dados

Quando adequado, os resultados de grupos de estudos comparáveis foram agrupados em metanálise, usando o modelo de efeitos randômicos como padrão. Para resultados dicotômicos, foram calculados a taxa de risco e o IC95%. Quando dois ou mais estudos apresentavam dados contínuos pelo mesmo instrumento de avaliação validado, usando as mesmas unidades de medida, os dados foram agrupados como DM com IC95%. Quando os estudos primários declararam a mesma variável, usando diferentes instrumentos e diferentes unidades de medida, usamos a diferença média padronizada com IC95%.

### Análise de subgrupos e investigação de heterogeneidade

Planejou-se a análise de subgrupos para os seguintes dados demográficos: idade (adolescentes, adultos e idosos acima de 65 anos); tipo de intervenção cirúrgica (ATJ); e início da reabilitação (paciente ambulatorial ou internado).

### Análise de sensibilidade

Foram planejadas análises de sensibilidade para medir os efeitos da inclusão de estudos sob risco de viés de seleção (ocultação de alocação inadequada ou incerta) ou viés de detecção (cegamento inadequado ou incerto do examinador dos resultados). Também foi planejada a avaliação da presença de viés de estudo de pequeno porte (por exemplo: o efeito da intervenção é mais benéfico em estudos de pequeno porte) na metanálise, comparando a estimativa de efeito fixo com a estimativa de efeitos aleatórios para resultados primários.

### Tabelas de ‘resumo de achados’ e avaliação da qualidade das evidências

Quando houver evidências suficientes no futuro para justificar a elaboração de tabelas de resumo de achados, elas serão criadas para comparações principais. Usamos a abordagem GRADE para avaliar a qualidade das evidências relacionadas a cada resultado importante listado nos tipos de medidas de resultado.^(17)^

## RESULTADOS

### Resultados da pesquisa

A estratégia de pesquisa (concluída em agosto de 2013) identificou um total de 584 registros nos seguintes bancos de dados atuais: *Cochrane Library* (129), PubMed (166), Embase (143), LILACS (130), Ensaios Clínicos (11) e *Controlled Trials* (5).

A pesquisa resultou na identificação de 18 estudos potencialmente elegíveis, dos quais recuperamos os documentos completos. Um total de quatro estudos, publicados entre 1994 e 2013, foram incluídos na revisão.^(18-21)^

Ao todo, quatro estudos foram incluídos; oito, excluídos; e seis estavam em andamento (Figura 1).

**Figura 1:** Diagrama do fluxo de estudo

### Estudos incluídos

Esta revisão baseou-se em quatro estudos clínicos randomizados: Gotlin et al.,^(18)^ Levine et al.,^(19)^ Petterson et al.^(20)^ e Stevens-Lapsley et al.^(21)^ (todos os estudos apresentados em inglês). Esses estudos foram encontrados no PubMed, *Cochrane Library* e Embase. Os detalhes dos estudos incluídos estão no anexo 2.

### Desenho do estudo

Gotlin et al.,^(18)^ Levine et al.,^(19)^ Petterson et al.^(20)^ e Stevens-Lapsley et al.^(21)^ foram estudos randomizados controlados de um único centro. Todos os estudos compararam dois grupos submetidos às mesmas intervenções (exercícios *versus* exercícios e EENM). Gotlin et al*.* ^(18)^ avaliaram exercícios e mobilização passiva contínua (Grupo Controle) comparado com exercícios, mobilização passiva contínua e EENM (grupo experimental).

### Participantes

Os quatro estudos incluídos totalizaram 376 participantes.

### Idade e sexo

Gotlin et al*.* ^(18)^ descreveram que a média de idade foi 64,8 anos no grupo de exercícios (controle) e 66,2 anos no grupo de exercícios mais EENM (intervenção). O Grupo Controle foi formado por 16 mulheres e 15 homens, e o grupo de intervenção por 20 mulheres e 15 homens. No estudo de Levine et al.,^(19)^ a média de idade foi de 65,1 anos no grupo controle, e 68,1 anos no grupo de intervenção. O Grupo Controle foi formado por 21 mulheres e 13 homens, e o grupo de intervenção, por 25 mulheres e 7 homens. Petterson et al.^(20)^ relataram que o Grupo Controle foi formado por 45 mulheres e 55 homens com média de idade de 65,2 anos. No grupo de intervenção, 47 participantes eram mulheres e 53 eram homens, apresentando média de idade entre os sexos de 65,3 anos. No estudo de Stevens-Lapsley et al.,^(21)^ a amostra consistia de 16 mulheres e 15 homens no Grupo Controle, e de 20 mulheres e 15 homens no grupo de intervenção, apresentando a média de idade entre os sexos de 64,8 anos para o Grupo Controle e de 66,2, para o grupo de intervenção.

Todos os participantes, em todos os estudos, tinham o diagnóstico de osteoartrose de joelho unilateral ou bilateral. Nenhum dos estudos descreveu a classificação ou ATJ prévia ao tratamento.

### Intervenções

Os estudos incluídos foram agrupados de acordo com as intervenções analisadas.

Gotlin et al.^(18)^ analisaram os efeitos de exercícios e mobilização passiva contínua (Grupo Controle) em comparação a exercícios, mobilização passiva contínua e EENM (grupo experimental) em 40 pacientes. Levine et al.^(19)^ compararam um programa de fortalecimento muscular e ganho de ADM do joelho *versus* EENM associada a apenas exercícios de ADM do joelho em 70 participantes. Petterson et al.^(20)^ e Stevens-Lapsley et al.^(21)^ compararam um programa de fortalecimento muscular progressivo (quadríceps) a EENM associada a fortalecimento muscular progressivo, ambos de início precoce (pós-operatório imediato), em 266 participantes.

### Resultados primários

#### Função ou deficiência

A função articular do joelho foi avaliada em três estudos.^(19-21)^ Levine et al.^(19)^ usou o *Timed Up and Go* (TUG), o questionário do Índice de Osteoartrose das Universidades *Western Ontario e McMaster* (WOMAC) e o *Knee Society Score* (KSS) para mensurar a função articular do joelho. Petterson et al.^(20)^ e Stevens-Lapsley et al.^(21)^ usaram testes validados − TUG, teste de escada (TE), e teste de caminhada de 6 minutos (TC6) − para avaliar o comprometimento funcional do joelho. Stevens-Lapsley et al.^(21)^ também usaram o questionário WOMAC. Além desses testes, Petterson et al.^(20)^ e Stevens-Lapsley et al.^(21)^ avaliaram a força muscular do quadríceps usando dinanometria.

#### Qualidade de vida

Gotlin et al.^(18)^ e Levine et al.^(19)^ não avaliaram esse desfecho. Petterson et al.^(20)^ e Stevens-Lapsley et al.^(21)^ avaliaram a qualidade de vida usando o SF-36.

#### Falha do tratamento

Nenhum dos estudos incluídos relatou falha do tratamento.

## Resultados secundários

### Dor

A dor foi avaliada em três estudos.^(19-21)^ Levine et al.^(19)^ usaram a escala validada do KSS para medir a dor, e Petterson et al.^(20)^ usaram uma escala validada (*Knee Outcome Survey -* KOS e *Activities of Daily Living Scale -* ADLS*)*; Stevens-Lapsley et al.^(21)^ mediram as queixas de dor individuais aplicando uma escala numérica visual.

### Amplitude de movimento

A ADM foi avaliada nos estudos de Gotlin et al.,^(18)^ Petterson et al.^(20)^ e Stevens-Lapsley et al.^(21)^ por meio de goniometria. Levine et al.^(19)^ também avaliaram a ADM do joelho dos participantes, mas não relataram qual instrumento foi utilizado para medir esse resultado.

## Estudos excluídos

Oito estudos foram excluídos por não satisfazerem os critérios de inclusão. Os motivos da exclusão desses estudos estão apresentados no quadro 1.

**Quadro 1:** Características dos estudos excluídos

Estudo	Motivo da exclusão
Petterson et al.^(22)^	Relato de caso
Mintken et al.^(23)^	Relato de caso
Stevens et al.^(9)^	Série de casos
Stevens-Lapsley et al.^(24)^	Estudo observacional
Bade et al.^(25)^	Revisão da literatura
Saleh et al.^(26)^	Revisão da literatura
Walls et al.^(27)^	Uso da EENM somente no pré-operatório
Stevens et al.^(28)^	Dados incompletos não publicados

EENM: estimulação elétrica neuromuscular.

## Estudos em andamento

Nossa pesquisa de estudos em andamentos resultou em 16 artigos da *Current Controlled Trials* e do Ensaios Clínicos. Dez estudos foram excluídos por não cumprirem nossos critérios de inclusão ou por serem irrelevantes. Foram incluídos: ISRCTN89785408, ISRCTN50117467, NCT01096524, NCT01548040, NCT00224913 e NCT01844193 (Anexo 3).

## Risco de viés nos estudos incluídos

Todos os estudos incluídos apresentavam falhas metodológicas que implicavam em moderado risco de viés (Figura 2).

**Figura 2:** Gráfico risco de viés

NMES: neuromuscular electrical stimulation. SD: standard deviation; IV: inverse variance; 95%CI: 95% confidence interval.

## Alocação (viés de seleção)

Gotlin et al.,^(18)^ Levine et al.,^(19)^ Petterson et al.^(20)^ e Stevens-Lapsley et al.^(21)^ não informaram como foi realizada a geração da sequência aleatória.

A ocultação da alocação antes da designação não foi descrita em nenhum dos estudos, impedindo sua avaliação (incerta).

## Cegamento (viés de condução e viés de detecção)

Todos os estudos foram considerados sob risco de viés de condução e de detecção. Como todos comparavam intervenções de fisioterapia, não foi possível cegar os provedores do tratamento. Nenhum estudo incluiu intervenções simuladas, e os participantes não foram cegados. Teria sido possível cegar os examinadores dos resultados, e apenas dois estudos mencionaram o cegamento dos examinadores.^(18-20)^

## Dados de resultados incompletos (viés de seguimento)

Os estudos em que 80% ou mais dos participantes concluíram o seguimento e aqueles em que houve perdas foram equilibrados entre os grupos de intervenção, sendo considerados com baixo risco de viés. Todos os estudos incluídos^(18-21)^ foram considerados com baixo risco de viés.

## Relatório seletivo (relatório de viés)

Três estudos^(19-21)^ foram considerados com baixo risco de viés, uma vez que seus protocolos e resultados pré-especificados estavam disponíveis. Por outro lado, o estudo de Gotlin et al.^(18)^ foi considerado com alto risco de viés, uma vez que não apresentou um protocolo específico.

## Outras fontes de viés em potencial

Os estudos de Gotlin et al.,^(18)^ Petterson et al.^(20)^ e Stevens-Lapsley et al.^(21)^ pareciam estar isentos de outros vieses. Somente o de Levine et al.^(19)^ foi considerado com alto risco de viés, uma vez que não apresentou um protocolo EENM.

## Efeitos das intervenções

Os estudos incluídos avaliaram os seguintes resultados: função articular ou deficiência, ADM, qualidade de vida e falha do tratamento. Outros resultados planejados em nosso protocolo não foram avaliados devido à insuficiência de dados.

### Comparação

Estimulação elétrica neuromuscular *versus* exercícios (com ou sem mobilização passiva contínua) com início na primeira semana de pós-operatório.

## Função ou incapacidade

As medidas de função articular foram analisadas e disponibilizadas na seguinte sequência: TUG, TC6, TE, ativação do quadríceps, força do quadríceps femoral e WOMAC.

***Timed Up and Go***

No estudo de Levine et al.,^(19)^ não houve diferenças significativas tanto no desfecho a curto prazo (DM: 1,32; IC95%: -0,45-3,09) quanto no de longo prazo (DM: 0,39; IC 95%: -1,02-1,80). No estudo de Stevens-Lapsley et al.,^(21)^ notamos uma diferença significativa favorecendo a EENM tanto no desfecho a curto prazo (DM: -2,00; IC95%: -3,27- -0,73) quanto no de longo prazo (DM: -1,60; IC95%: -2,86- -0,34). O agrupamento de dados dos estudos não mostrou nenhuma diferença estatisticamente significativa entre os dois grupos de intervenção - EENM e exercícios (DM: -0,87; IC95%: -1,90-0,17), (DM: -0,72; IC95%: -1,66-0,22) no desfecho de curto e de longo prazo, respectivamente (Figura 3).

**Figura 3:** Gráfico *Timed Up and Go*

NMES: neuromuscular electrical stimulation. SD: standard deviation; IV: inverse variance; 95%CI: 95% confidence interval.

Petterson et al.^(20)^ não relataram nenhuma diferença estatisticamente significativa (p>0,08).

### Teste de caminhada de 6 minutos

A diferença entre os grupos no estudo de Stevens- Lapsley et al.^(21)^ favoreceu marginalmente o Grupo Controle no desfecho a curto prazo (DM: 62,60; IC95%: 15,51-109,69). No desfecho a longo prazo, nenhuma diferença entre os grupos foi observada (DM: 46,80; IC95%: -0,53-94,13). Nenhuma diferença estatisticamente significativa foi relatada por Petterson et al.^(20)^ (p>0,08).

### Teste de escada

Petterson et al.^(20)^ não relataram diferença significativa para este resultado no desfecho a curto e longo prazo (p>0,08). No estudo de Stevens-Lapsley et al.^(21)^, nenhuma diferença estatisticamente significativa (DM: -2,70; IC95%: -6,40-1,00), (DM: -3,30; IC95%: -7,27-0,67) foi observada no desfecho a curto prazo e a longo prazo, respectivamente.

### Ativação do quadríceps (%)

Petterson et al.^(20)^ relataram o mesmo, com p>0,08. Stevens-Lapsley et al.^(21)^ não relataram diferença estatisticamente significativa no desfecho a curto prazo (DM: 1,10; IC95%: -5,13-7,33) entre os dois grupos e no desfecho pós-ATJ a curto prazo (DM: 1,70; IC95%: -4,17-7,57) (Figura 4).

**Figura 4:** Gráfico ativação do quadríceps

### Força do quadríceps femoral (N-m/kg)

Petterson et al.^(20)^ também relataram a ausência de diferença estatisticamente significativa entre os grupos (p>0,08). Nenhuma diferença estatisticamente significativa entre os dois grupos no desfecho a curto prazo (DM: 0,22; IC95%: -0,02-0,46) e no desfecho a longo prazo (DM: 0,16; IC95%: -0,09-0,41) foi observada no estudo de Stevens-Lapsley et al.^(21)^

## Qualidade de vida

O questionário SF-36 foi usado para avaliar a qualidade de vida durante e após a intervenção de fisioterapia, tanto no estudo de Petterson et al.^(20)^ quanto no de Stevens-Lapsley al.,^(21)^ e os dados foram analisados de acordo com seus componentes.

### Resumo do componente físico do SF-36

Petterson et al.^(20)^ não relataram diferença significativa entre os grupos (p>0,08). O estudo de Stevens-Lapsley et al.^(21)^ também não relatou diferença significativa a curto prazo (DM: 3,20; IC95%: -1,50-7,90) e a longo prazo (DM: 1,90; IC95%: -1,97-5,77).

### Resumo do componente mental do SF-36

Petterson et al.^(20)^ não relataram diferença significativa, com p>0,08. No resumo do componente mental, Stevens-Lapsley et al.^(21)^ também não relataram uma diferença significativa, tanto a curto (DM: 3,70; IC95%: -1,55-8,95) quanto a longo prazo (DM: 3,00; IC95%: -0,39-6,39).

### Qualidade de vida e função articular

O questionário WOMAC foi usado por Levine et al.^(19)^ para avaliar a função articular do joelho, sem diferenças estatisticamente significativas entre os grupos, tanto no desfecho a curto (DM: 5,67; IC95%: -1,88-13,22) quanto a longo prazo (DM: 5,81; IC95%: -3,01-14,63). Stevens- Lapsley et al.^(21)^ também não relataram diferença estatisticamente significativa nos desfechos a curto (DM: -6,80; IC95%: -15,04-1,44) e longo prazo (DM: -4,30; IC95%: -9,99-1,39). O agrupamento de dados não mostrou diferença estatisticamente significativa tanto no desfecho a curto prazo (DM: -0,02; IC95%: -5,59-5,54) quanto a longo prazo (DM: -1,33; IC95%: -6,11-3,45) entre os dois grupos de intervenção.

Levine et al.^(19)^ usaram o questionário KSS para avaliar a função articular do joelho e não relataram diferença estatisticamente significativa, tanto no desfecho a curto prazo (DM: 4,97; IC95%: -4,65-14,59) quanto a longo prazo (DM: 7,92; IC95%: -2,29-18,13).

Petterson et al.^(20)^ usaram o questionário KOS para avaliar a função articular do joelho e não relataram diferença estatisticamente significativa nos desfechos a curto e longo prazo (p>0,01).

## Falha do tratamento

Nenhum dos estudos incluídos relatou falha do tratamento.

## Dor

Nenhum dos estudos incluídos avaliou esse desfecho individualmente.

## Amplitude de movimento

### Flexão do joelho

O estudo de Levine et al.^(19)^ não relatou nenhuma diferença significativa a curto prazo (DM: -3,20; IC95%: -8,52-2,12) e a longo prazo (DM: 2,30; IC95%: -4,25 -8,85). O estudo de Stevens-Lapsley et al.^(21)^ não relatou nenhuma diferença estatisticamente significativa entre os grupos - EENM e exercícios (DM: 2,90; IC95%: -1,89-7,69 e DM: 2,40; IC95%: -1,82-6,62) no desfecho a curto prazo e a longo prazo, respectivamente. Petterson et al.^(20)^ também não relataram nenhuma diferença estatisticamente significativa (p>0,01). O agrupamento de dados dos estudos não mostrou nenhuma diferença estatisticamente significativa entre os dois grupos de intervenção (DM: 0,17; IC95%: -3,39-3,73 e DM: 2,37; IC95%: -1,18-5,92) tanto no desfecho de curto prazo quanto no de longo prazo, respectivamente.

### Extensão do joelho

Gotlin et al.^(18)^ e Stevens-Lapsley et al.^(21)^ relataram uma diferença estatisticamente significativa favorecendo o grupo de intervenção em relação ao sem intervenção no desfecho a curto prazo (DM: -2,65; IC95%: -4,05- -1,25 e longo prazo DM: -2,40; IC95%: -4,09- -0,71), sem nenhuma diferença estatisticamente significativa, a qual foi relatada somente no estudo de Stevens-Lapsley et al.^(21)^ (DM: -0,60; IC95%: -2,43-1,23). Levine et al.^(19)^ não relataram diferenças significativas tanto no desfecho a curto (DM: 0,65; IC95%: -2,00-3,30) quanto no longo prazo (DM: -0,96; IC95%: -3,62-1,70). Quando os dados foram agrupados, uma diferença estatisticamente significativa foi observada no desfecho a curto prazo (DM: -2,09; IC95%: -3,09- -1,09); contudo, não houve diferença estatisticamente significativa no desfecho a longo prazo (DM: -0,72; IC95%: -2,22-0,79) em ambos os grupos de intervenção.

Petterson et al.^(20)^ não relataram diferença significativa entre os dois grupos durantes todos os meses de intervenção (p>0,01).

## DISCUSSÃO

Nesta revisão, somente foram incluídos estudos clínicos randomizados, resultando na análise de quatro estudos considerados de risco de viés moderado e na avaliação das intervenções de fisioterapia cinesioterápica comparadas com o uso da EENM com fisioterapia em 376 participantes submetidos à ATJ.

Nenhuma evidência indicou que a EENM com fisioterapia proporcionou benefícios referentes à qualidade de vida. O tratamento pós-operatório com EENM pode melhorar a função do quadríceps femoral, mas não estamos seguros quanto à efetividade dessa intervenção, devido à baixa qualidade da evidência.

Evidências muito baixas dos estudos incluídos apresentaram qualidade geral baixa resultante de falhas metodológicas, incluindo a ausência da ocultação da alocação, e do cegamento dos participantes e da equipe em todos os estudos. No entanto, os resultados quantitativos desta revisão devem ser cuidadosamente interpretados, exigindo-se a confirmação desses dados por evidência decorrente de estudos de alta qualidade metodológica.

Acreditamos que nossa estratégia de pesquisa foi completa, sem restrições de idioma. No entanto, é possível que tenhamos deixado de identificar estudos potencialmente elegíveis. Tentamos contatar os autores dos estudos incluídos a fim de obter alguns dados, mas não obtivemos sucesso.

Encontramos uma revisão sistemática que comparava a EENM com a terapia com exercícios para tratar a inibição do quadríceps após ATJ (Monaghan et al.),^(29)^incluindo dois estudos clínicos randomizados e não randomizados, com um total de 69 indivíduos. Os resultados de nosso estudo foram condizentes com os resultados da revisão sistemática Cochrane, e nossa conclusão foi semelhante à daquela publicação.

Monaghan et al.^(29)^ avaliaram o uso da EENM para fortalecimento do quadríceps pré- e pós-ATJ e relataram não ter havido diferenças significativas para esses desfechos; consideraram, porém, os estudos incluídos com alto risco de viés devido às limitações do projeto do estudo e por terem apresentado imprecisão nos resultados, impedindo a realização de uma metanálise. Os autores concluíram que os estudos identificados não permitiam nenhuma definição no tocante à EENM pré- ou pós-ATJ.

Monaghan et al.^(29)^ também interpretaram que os participantes submetidos à EENM do quadríceps apresentaram discreta vantagem na melhoria da função e menos deficiência do que os que foram tratados de modo conservador no seguimento de curto prazo, conforme observamos na prática clínica e de modo condizente com os achados de Gotlin et al.^(18)^ No entanto, a eficácia foi reduzida no seguimento a longo prazo.

Portanto, esta revisão é inconclusiva no tocante à eficácia da EENM, e mais evidências são necessárias para apoiar ou refutar seu uso na ativação do quadríceps após ATJ. Os autores estão cientes de que este assunto de revisão é alvo de uma investigação em andamento e que pode ser atualizado a fim de incorporar novas evidências.

## CONCLUSÃO

As evidências muito baixas provenientes dos estudos incluídos encontrados nesta revisão não permitiram quaisquer conclusões referentes à aplicação da estimulação neuromuscular para fortalecimento do quadríceps com fisioterapia, antes ou depois da artroplastia total de joelho. Até o momento, as evidências para o uso da estimulação para o fortalecimento do quadríceps, neste grupo de pacientes, são incertas. No entanto, é essencial que estudos futuros verifiquem a força do quadríceps pré- e pós-estimulação neuromuscular usando avaliações confiáveis e ferramentas validadas, além de uma clara descrição da dose aplicada no projeto do estudo. Também é de grande importância que os resultados sejam apresentados adequadamente para a realização da metanálise.
